# Canine bilateral zygomatic sialadenitis: 20 cases (2000‐2019)

**DOI:** 10.1111/jsap.13844

**Published:** 2025-03-23

**Authors:** A. E. Enache, S. Maini, M. Pivetta, E. Jeanes, L. Fleming, C. Hartley, R. Tetas Pont

**Affiliations:** ^1^ North Downs Specialist Referrals Bletchingley UK; ^2^ Department of Clinical Sciences and Services Queen Mother Hospital for Animals, Royal Veterinary College (RVC) London UK; ^3^ Antech Imaging Services Irvine California USA; ^4^ Dick White Referrals, Station Farm Six Mile Bottom UK; ^5^ Royal (Dick) School of Veterinary Studies University of Edinburgh Edinburgh UK

## Abstract

**Objectives:**

To describe clinical findings, cross‐sectional imaging features, management and outcome of dogs with bilateral zygomatic sialadenitis.

**Materials and Methods:**

Clinical databases of three referral institutions were searched for dogs diagnosed with bilateral zygomatic sialadenitis who underwent magnetic resonance imaging or computed tomography of the head. Signalment, history, clinical, laboratory and imaging findings were reviewed.

**Results:**

Twenty dogs with a mean age (±SD) of 7.1 (±2.7) years were included; Labradors were overrepresented (10/20). Common clinical signs included pain on opening the mouth (18/20), conjunctival hyperaemia (16/20), exophthalmos (15/20), periorbital pain (15/20), third eyelid protrusion (11/20) and resistance to retropulsion of the globes (11/20). Fifteen of twenty dogs had at least one concurrent systemic disease: skin allergy (5/15), hypertension (3/15), gastrointestinal (3/15), kidney (3/15), neurological (3/15) and periodontal disease (2/15), pancreatitis (2/15) and neoplasia (2/15). Neutrophilia (9/18) and leukocytosis (7/18) were the most common haematological abnormalities. When performed (11/20), aspiration cytology revealed predominantly degenerate neutrophils (9/11) and only 2/9 culture samples yielded bacterial growth. The zygomatic glands were predominantly hyperintense on both T1 and T2‐weighted images (22/24) and symmetrically enlarged (20/24) with marked and heterogeneous contrast enhancement (18/24). In the computed tomography studies, the zygomatic glands were all hyperattenuating and contrast enhancing. Treatment included systemic antimicrobial (18/20), anti‐inflammatory (14/20) and supportive treatment (16/20). Clinical signs improved in 16/20 dogs; however, 4/20 dogs were euthanised due to severe systemic disease.

**Clinical Significance:**

Bilateral zygomatic sialadenitis is frequently associated with systemic disease in dogs. Clinical signs generally improve with systemic antimicrobial, anti‐inflammatory and supportive treatment.

## INTRODUCTION

The zygomatic gland, also referred to as the orbital or dorsal buccal gland, is a salivary gland specific to carnivores (Howard & de Lahunta, 2013). It has a globular to pyramidal shape with the base directed dorsally and caudally, and it occupies the lateral two‐thirds of the orbital floor ventromedial to the zygomatic arch, dorsal and lateral to the pterygoid muscles (Howard & de Lahunta, 2013). The zygomatic gland has one major and two to four minor ducts that open on a papilla near the vestibular fornix located approximately 1 cm caudal to the parotid papilla and lateral and caudal to the superior first molar tooth (Howard & de Lahunta, 2013). The zygomatic gland receives parasympathetic innervation via the glossopharyngeal nerve and vascular supply from branches of the maxillary artery (Howard & de Lahunta, 2013).

Salivary gland disease appears to be rare or underreported, with no current studies describing its prevalence in dogs. In one histopathological study, salivary gland samples (healthy and diseased) accounted for 0.3% of the total caseload over 3 years (Spangler & Culbertson, 1991). Reported conditions affecting the salivary glands include: phenobarbital‐responsive sialadenosis, neoplasia, necrotizing sialometaplasia, sialocoele, salivary gland infarction, sialolithiasis and sialadenitis (Alcoverro et al., 2014; Boland et al., 2013; Cannon et al., 2011; Kelly et al., 1979; Lee et al., 2014; Martinez et al., 2018; Mason et al., 2014; McGill et al., 2009; Schroeder & Berry, 1998; Spangler & Culbertson, 1991).

Both sialadenosis and sialadenitis are diseases characterised by enlargement of the salivary glands. Phenobarbital‐responsive sialadenosis is an idiopathic disease in dogs characterised by bilateral painless, non‐inflammatory, uniform and nonneoplastic enlargement of the salivary glands, mainly submandibular glands, often associated with retching and gulping (Alcoverro et al., 2014; Boydell et al., 2000). The aetiology of this condition remains unknown; however, it has been associated with an unusual form of limbic epilepsy. Therefore, a diagnosis of phenobarbital‐responsive sialadenosis is often made by exclusion of other diseases with similar signs in addition to the typical clinical signs (painless bilateral enlargement of the glands) and the rapid response to phenobarbital treatment alone (Alcoverro et al., 2014, Boydell et al., 2000). Sialadenitis refers to an inflammatory enlargement of the salivary glands often accompanied by pain, swelling and other clinical signs of an inflammatory response with evidence of inflammatory infiltration on cytological or histopathological samples, typically responsive to antimicrobial and anti‐inflammatory treatment. However, sialadenitis has also been linked to afferent vagal impulses and limbic epilepsy in a subset of dogs that responded to treatment with phenobarbital (Martinez et al., 2018). There are reports of phenobarbital treatment in dogs with sialadenitis (Martinez et al., 2018; McGill et al., 2009; Schroeder & Berry, 1998). More recently, Martinez et al. (2018) described rapid clinical response to phenobarbital in a dog diagnosed cytologically with bilateral sialadenitis; hence the importance of considering the clinical picture, cytological/histological and imaging findings.

Clinical reports describing zygomatic sialadenitis are infrequent, with several proposed aetiologies such as trauma, secondary to retrobulbar disease, ascending oral or systemic infection and presumed immune‐mediated (Boland et al., 2013; Cannon et al., 2011; Fischer et al., 2018; Martinez et al., 2018; McGill et al., 2009; Spangler & Culbertson, 1991). Affected dogs usually present with signs of a space‐occupying, extraconal orbital lesion such as third eyelid protrusion, exophthalmos, different degrees of impaired retropulsion and pain on opening the mouth (Boland et al., 2013; Cannon et al., 2011; Martinez et al., 2018; McGill et al., 2009).

Reports of dogs with zygomatic sialadenitis of bilateral presentation are even more sparse, with only nine cases described in the literature (Bailey et al., 2021; Cannon et al., 2011; Fischer et al., 2019; Martinez et al., 2018; McGill et al., 2009; Winer et al., 2018). Little information is available regarding the clinical presentation, imaging features, concurrent systemic disease, treatment and outcome of such cases. The purpose of this retrospective study was to describe the clinical findings, cross‐sectional imaging features, management and outcome of dogs diagnosed with bilateral zygomatic sialadenitis (BZS).

## MATERIALS AND METHODS

### Study design and inclusion criteria

Medical records of dogs referred to three referral centres, Queen Mother Hospital for Animals Royal Veterinary College (RVC), Animal Health Trust (AHT) and Langford Vets Small Animal Referral Hospital (LVH) in the United Kingdom from January 2000 to December 2018 (at the AHT) and December 2019 (at the RVC and LVH) for which imaging investigations indicated a diagnosis of BZS were reviewed. The processes governing the collection and use of data were approved by the Animal Health Trust (AHT ethical approval: 32‐2017), Royal Veterinary College (RVC ethical approval URN SR2017‐1182) and University of Bristol (VIN/18/002).

Firstly, at the AHT, medical records were searched by three investigators (AEE, SM and EJ), using the hospital's software medical records search function (Smartform). The records were initially searched by species (canine) and “zygomatic sialadenitis”. Then each individual case had its paper medical record manually searched in the institution's archives and subsequently reviewed.

At the RVC, the search was conducted by AEE via VetCompass programme, followed by searching each medical record on the RVC medical record software (CRIS). Several searches were made in the VetCompass programme including “bilateral zygomatic”, “bilateral zygomatic sialadenitis”, “bilateral zygomatic sialoadenitis”, “zygomatic sialoadenitis”, “zygomatic sialadenitis”, “zygomatic adenitis” and “necrotising sialoadenitis”.

At the LVH, patient data was searched by SM via the hospital's software RX Works combined with searching the hospital's imaging database. Each case was then carefully selected after reviewing their medical records.

Inclusion criteria comprised all cases with bilateral zygomatic gland enlargement consistent with inflammation (based on clinical signs of painful bilateral zygomatic gland enlargement, cytological or histological evidence of inflammation or cross‐sectional imaging evidence of inflammation such as surrounding cellulitis, myositis) that underwent either magnetic resonance imaging (MRI) or computed tomography (CT) studies.

Following patient selection, each case had its cross‐sectional imaging findings reviewed by MP for confirmation and further characterisation of the affected glands.

Patient information collected in a spreadsheet (Microsoft Excel) included age, gender, breed, duration of clinical signs prior to referral, primary complaint, department to which the case was referred history, clinical examination findings, visual status, blood results when available, CT or MRI findings, cytology, histopathology, culture and sensitivity results, treatment and outcome when available.

Samples for cytological and/or bacteriological testing were collected either via oral drainage through the pterygopalatine fossa or ultrasound‐guided fine‐needle aspirates through the temporal muscles. Results of the histopathological analysis of the globe and orbital contents or salivary glands were recorded when available.

For each dog, the outcome was determined based on information found in the clinical records following diagnosis.

### Diagnostic imaging investigations

Head magnetic resonance T1 and T2‐weighted images, pre‐ and post‐contrast or CT soft tissue and bone window images pre‐ and post‐contrast from the three referral centres were reviewed by MP, a Diplomate of the European College of Diagnostic Imaging. Parameters evaluated for each zygomatic gland included:

1. the shape of the gland: teardrop‐shaped, ovoid, pyramidal (to assess changes in the gland's shape with inflammation);
2. symmetry between the glands;
3. the margins of the glands: clearly defined, ill defined, smooth or irregular;
4. homogeneity of the gland parenchyma: homogeneous or heterogeneous (to assess how the inflammation affects the gland's parenchyma);
5. contrast enhancement: present or absent;
6. homogeneity of contrast uptake: homogeneous, heterogeneous, ring‐like (to assess the contrast uptake by the inflamed gland's parenchyma).

When applicable, the signal intensity of the glands on T1 and T2‐weighted images was classified as hypointense, isointense or hyperintense when its brightness was inferior, equal or superior to the signal of the cerebral cortex, respectively. The presence of surrounding cellulitis, myositis and mandibular and retropharyngeal lymphadenopathy was also recorded.

## RESULTS

### Signalment and clinical findings

Following the medical records search at the AHT, nine dogs with BZS were included after reviewing diagnostic imaging findings. At the LVH, the medical records search identified three cases of BZS that were subsequently included in the study.

At the RVC, the combined search on the VetCompass programme identified 134 cases, of which 13 cases were unilateral zygomatic sialadenitis, 3 cases of sialadenosis, 2 sialoliths, 1 sialocele, leaving 9 cases of BZS. Following the diagnostic imaging review, one case was excluded based on the strong suspicion of masticatory myositis as the diagnosis.

Among the three institutions, 20 dogs met the inclusion criteria: nine cases diagnosed at the AHT, eight cases at the RVC and three cases at the LVH. The mean age (±SD) was 7.1 (±2.7) years at presentation and there were 14 males (6 entire) and 6 females (1 entire). Labrador Retrievers represented half of the study population (10/20, 50%; Table 1).

**Table 1:** Signalment of the 20 dogs diagnosed with bilateral zygomatic sialadenitis, the specialist service to which they were initially referred to, presence of concurrent systemic signs/disease, duration of clinical signs prior to referral and most common clinical signs at presentation

Case	Signalment (breed, age, sex)	Referral service	Systemic disease	Referring/presenting complaint	Duration clinical signs prior to referral (days)	Exophthalmos	Conjunctival hyperaemia	Periorbital pain	Pain on opening the mouth	Resistance to retropulsion	Third eyelid protrusion
1	Labrador, 8 years 3 months, male neutered	O	No	Lethargy, inappetence, vomiting, hypersalivation, red and puffy eyes, pain on opening the mouth	3	Yes	Yes	Yes	Yes	–	Yes
2	Labrador, 7 years 9 months, male	O	No Previous history of allergic skin disease	Left third eyelid and temporal swelling, painful on opening mouth	2	Yes	Yes	Yes	Yes	Yes	Yes
3	Labrador, 8 years 4 months, male neutered	IM	Acute renal injury, tertiary hyperparathyroidism, hypertension	Difficult to eat dry food, exophthalmos	14	Yes	Yes	–	Yes	Yes	–
4	Labrador, 8 years 3 months, female neutered	O	Diabetes mellitus (controlled)	Inappetence, red eyes	1	Yes	Yes	Yes	Yes	Yes	Yes
5	Crossbreed, 9 years 8 months, male	IM	Kidney disease, pancreatitis, hypertension, chronic leishmaniasis, mild compressive cervical mielopathy	Collapsing episodes, vomiting	3	No	No	No	No	No	No
6	Labrador, 3 years 6 months, female	IM	Proximal renal tubular acidosis, suppurative nasopharyngitis	Lethargy, polyuria, polydipsia, vomiting, anorexia, conjunctivitis, swollen and painful face, pain on opening the mouth	4	Yes	Yes	Yes	Yes	No	–
7	Labrador, 3 years 4 months, female neutered	N	No	Periorbital swelling, pain on opening mouth	2	–	–	Yes	Yes	–	–
8	Jack Russel terrier, 8 years 3 months, male neutered	O	Gastrointestinal foreign body, sepsis	Polyuria, polydipsia, intermittent vomiting, inappetence, exophthalmos	7	Yes	Yes	Yes	Yes	Yes	–
9	Cavalier King Charles Spaniel, 4 years 1 month, male neutered	N	Pancreatitis, herniation of the cerebellar vermis Chiari‐type 1	Lethargy, hyporexia, diarrhoea, marked ocular and oral pain, bilateral exophthalmos	7	Yes	Yes	Yes	Yes	Yes	Yes
10	Staffordshire Bull terrier, 7 years 1 month, male	ECC	Right‐sided facial nerve paresis associated to peripheral vestibular disease	Progressive left periocular and third eyelid swelling, intermittent appetite	10	Yes	Yes	Yes	Yes	–	Yes
11	Crossbreed, 12 years 2 months, male neutered	ECC	No	Anorexia, regurgitation, diarrhoea, lethargy, swollen eyes, third eyelid protrusion	3	Yes	Yes	–	Yes	Yes	Yes
12	Labrador, 6 years 4 months, male	ECC	Neutrophilic vasculitis, anaphylaxis, hypertension, skin disease	Breathing abnormalities, weakness, anaphylaxis, diarrhoea, inflamed eyes	1	Yes	Yes	Yes	Yes	–	Yes
13	Labrador, 11 years, female neutered	IM	Hypothyroidism, skin disease	Haemorrhagic gastro‐enteritis, acute pain on opening jaw and bilateral periocular swelling	5	No	Yes	Yes	Yes	Yes	–
14	French bulldog, 8 years 4 months, male neutered	N	Intraventricular neoplasia	Lethargy, ataxia, anorexic, intermittent pain, aggression, inappropriate urination	7	–	–	Yes	–	–	–
15	Labrador 6 years 5 months, male	IM	Skin disease, duodenal foreign body	Lethargy, vomiting, pyrexia, third eyelid protrusion	6	Yes	Yes	Yes	Yes	Yes	Yes
16	Cavalier King Charles Spaniel, 4 years 10 months, male neutered	IM	Rhinitis, tonsillitis, nasopharyngitis, degenerative mitral valve disease	Reverse sneezing/choking episodes, intermittent diarrhoea	1	–	–	Yes	Yes	–	–
17	Labrador, 5 years, female neutered	IM	Skin disease	Weight loss, skin infections, polydipsia, facial swelling, pain on opening the mouth	1	Yes	Yes	–	Yes	Yes	Yes
18	Wire‐haired fox terrier, 7 years 7 months, male	IM	Skin and periodontal disease	Lethargy, inappetence, pyrexia, facial pain	4	Yes	Yes	Yes	Yes	Yes	–
19	Rough Collie, 2 years, female neutered	IM	No	Acute onset vomiting prior to onset of facial swelling	5	Yes	Yes	Yes	Yes	–	Yes
20	Jack Russel terrier, 9 years 8 months, male neutered	O	Lymphoma, maxillary osteomyelitis, periodontal disease	Lethargy, vomiting, dry cough, third eyelid protrusion and swelling	3	Yes	Yes	–	Yes	Yes	Yes

ECC Emergency and Critical Care department, IM Internal Medicine department, N Neurology department, O Ophthalmology

Most cases (9/20) were referred to the Internal Medicine service, whereas only 5/20 cases were referred to the Ophthalmology service, 3/20 to the Neurology service and 3/20 to the Emergency and Critical Care services among the three referral centres where applicable.

**FIG 1:**
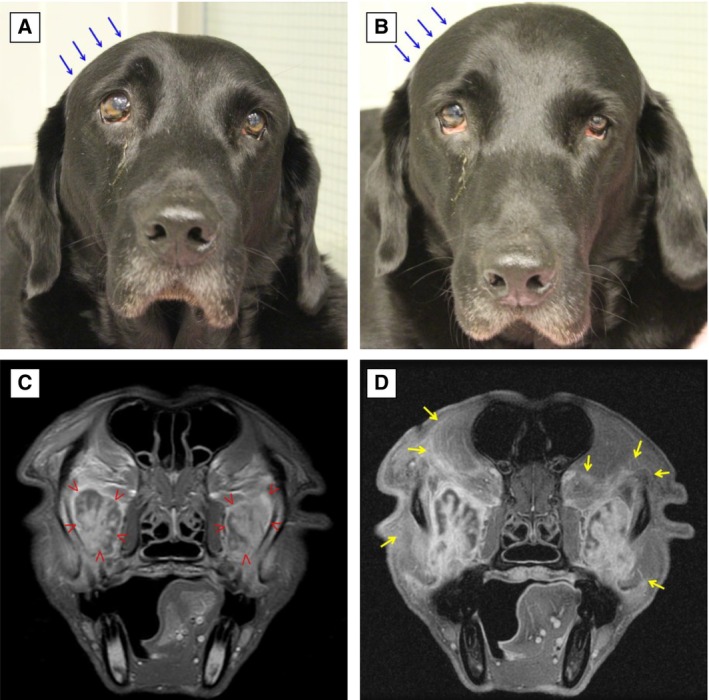
Clinical presentation and magnetic resonance imaging (MRI) findings of an 8‐year‐3‐month‐old male neutered Labrador retriever (Case 1) diagnosed with bilateral zygomatic sialadenitis: (A, B) Frontal views. Note the soft tissue swelling overlying the right temporal and masseter muscles (blue arrows), mild bilateral third eyelid protrusion and dorsolateral deviation of the right globe. (C) T1‐weighted (T1W) MRI post‐contrast and (D) 3D. SPGR post contrast fat suppression showing bilateral swollen and hyperintense zygomatic glands (red arrowheads) with rounded margins, and heterogenous contrast enhancement (arborizing pattern). Patchy contrast enhancement is also appreciated on the bordering fascial planes of the temporal and masseter muscles (yellow arrows).

**FIG 2:**
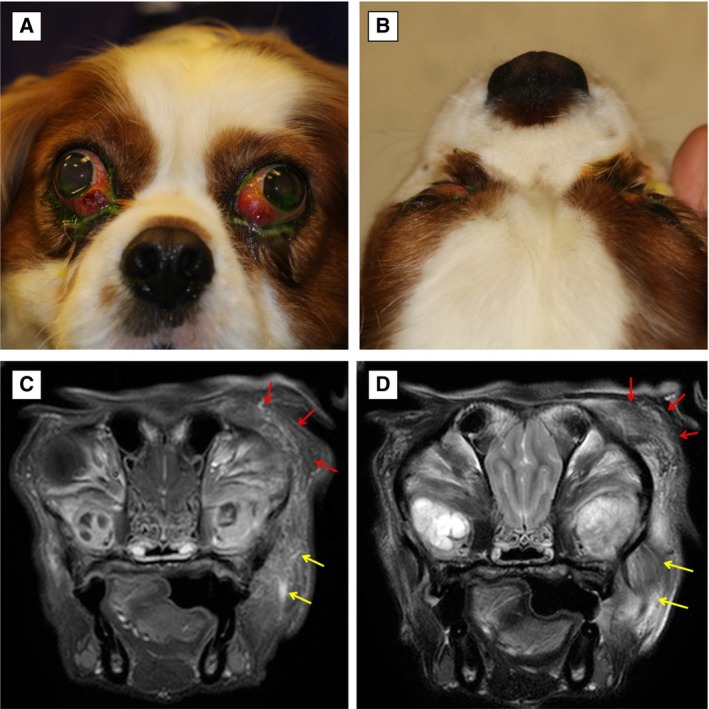
Frontal (A) and dorsoventral (B) views of a 4‐year‐1‐month‐old male neutered Cavalier King Charles Spaniel at presentation (Case 9). Note the marked bilateral exophthalmos, dorsolateral globe deviation, third eyelid protrusion and conjunctival hyperaemia. There was a paraxial ventral superficial corneal ulcer in both eyes due to the corneal exposure (lagophthalmos), which have been stained with fluorescein dye. (C) Post‐contrast T1W and (D) T2‐weighted (T2W) transverse MRI of Case 9. Note the enlarged zygomatic glands with a patchy contrast uptake on the T1‐W image and diffusely hyperintense on T2W. The left masseter (yellow arrows) and temporalis (red arrows) muscles reveal streaky contrast enhancement.

**FIG 3:**
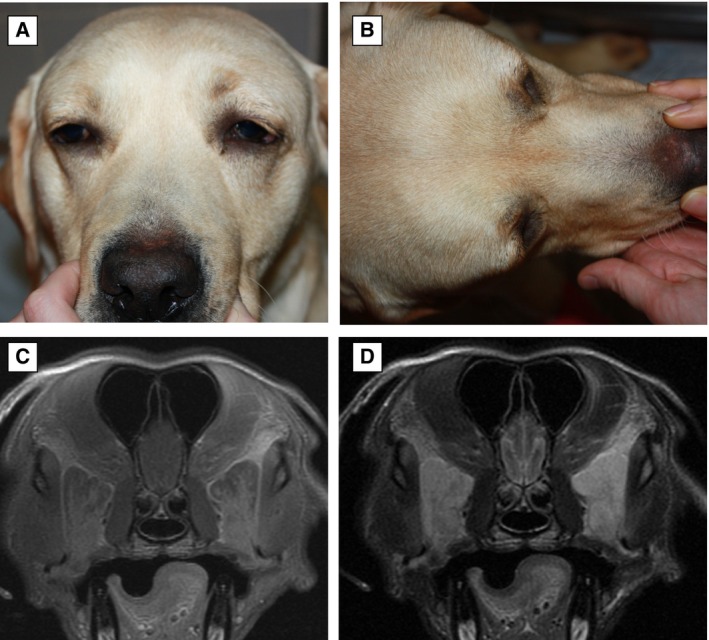
Frontal (A) and dorsoventral (B) views of a 3‐year‐6‐month‐old female entire Labrador retriever at presentation (Case 6). Note bilateral marked swelling overlying the temporal muscles, pain upon palpation of the head, bilateral exophthalmos and mild soft tissue swelling ventral to the lower eyelids. (C) Post‐contrast T1W and (D) T2W transverse MRI of Case 6 bilateral swollen zygomatic glands with rounded margins, heterogenous contrast enhancement and diffusely hyperintense signal on T2W image.

**FIG 4:**
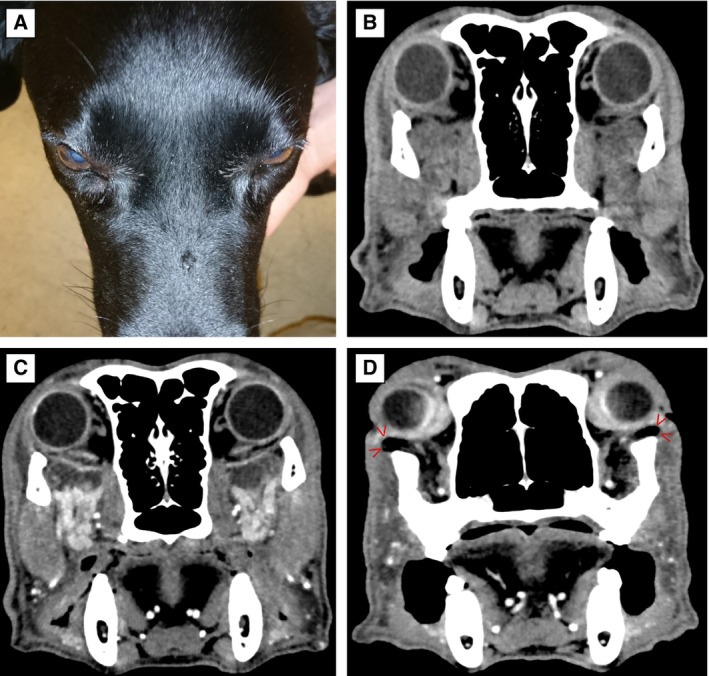
Eleven‐year‐old female neutered Labrador retriever (Case 13) presented with severe pain on opening of the mouth and only subtle soft tissue swelling ventral to the lower eyelids. (A) Soft‐tissue window computed tomography (CT) images pre‐contrast (B) and (C) post‐contrast reveal that both zygomatic salivary glands are enlarged, hyperattenuating with marked and heterogeneous contrast enhancement. (D) Soft tissue window CT post‐contrast. The orbital fat ventral to the lower eyelid margins appears prolapsed laterally (red arrowheads) due to the mass effect of the enlarged zygomatic glands.

**FIG 5:**
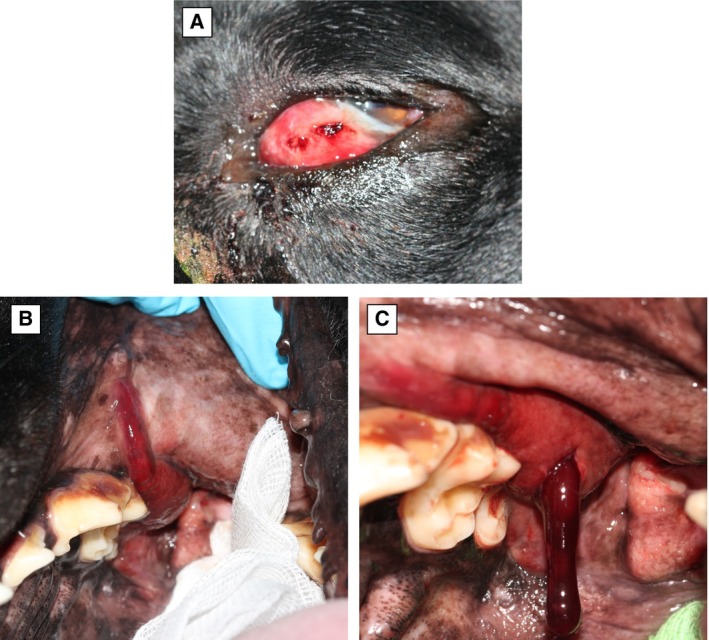
Clinical appearance and close‐up images of the oral cavity in a 7‐year‐9‐month‐old male entire Labrador retriever (Case 2), presented with bilateral zygomatic sialadenitis. (A) Note the marked periorbital swelling, third eyelid protrusion, chemosis and hyperaemia of third eyelid conjunctiva, mucoid discharge and dorso‐lateral deviation of the eye globe. (B) Note the hyperaemia and swelling of the oral mucosa overlying the pathway of the zygomatic duct. (C) Oral access to the orbital space through the inflamed pterygopalatine fossa in the same case. Note the presence of copious amount of tenacious haemorrhagic fluid admixed with saliva draining from the surgical incision.

Commonly reported clinical signs (Table 1) included pain‐related symptoms, such as pain on opening the mouth (18/20), pain on palpation of the head/periocular area (15/20) and resistance to retropulsion of the globe (11/20). Ocular changes (Table 1) included conjunctival hyperaemia (16/20), unilateral/bilateral exophthalmos (15/20), periocular/facial swelling (12/20) and third eyelid protrusion (11/20) (Figs 1, 2, 3, 4). Other ocular findings included chemosis (6/20), corneal ulceration (4/20 dogs, six eyes), anterior uveitis (3/20), blepharospasm (3/20), ocular discharge (3/20), lower lid ectropion (1/20), eyelid mass (1/20) and vitreal haemorrhage (1/20). On oral examination, the buccal mucosa overlying the openings of the zygomatic gland duct was recorded to be hyperaemic and swollen in 4/20 cases (Fig 5).

At presentation, 14/20 dogs were visual in both eyes, 3/20 were unilaterally blind, 1/20 was bilaterally blind and in 2/20 cases, the visual status was not reported.

The average duration of clinical signs before referral was 4.4 days (minimum 1 day and maximum 14 days).

Concurrent systemic disease at diagnosis and following investigations was present in 15/20 cases (Table 1), having at least one of the following: skin allergy (5/15), hypertension (3/15), gastrointestinal (3/15), kidney (3/15), neurological (3/15) and periodontal disease (2/15), pancreatitis (2/15) and neoplasia (2/15). At referral, lethargy was reported in 9/20 dogs and gastrointestinal signs were present in 13/20 dogs (vomiting and diarrhoea in 8/20 and in 5/20 dogs respectively). Ten of 20 dogs were reported to have anorexia/hyporexia or difficulty eating and 2/20 dogs had respiratory signs (coughing and reverse sneezing). 4/20 dogs had a body temperature over 39.2°C. Hypersalivation was reported in 3/20 dogs.

BZS was diagnosed incidentally following further investigations in three cases (Cases 5, 14 and 16) that were referred for other reasons (Table 1). Case 5 was referred to the Internal medicine department with a history of acute onset vomiting and generalised ataxia, also having a long‐standing history of leishmaniasis and azotaemia. Neurological examination was consistent with multifocal neuro‐localisation with potential involvement of the forebrain, vestibular/vestibulo‐cerebellar system and C1–C5 spinal cord segments, a cerebrovascular event being considered most likely. Following initial investigations, the dog was diagnosed with proteinuric kidney disease with hypertension, gastritis, mild abdominal effusion, pancreatitis and a hypoechoic lesion associated with the pancreas. Subsequently, an MRI scan of the head revealed compressive myelopathy C6–C7 and bilateral moderate zygomatic gland enlargement with uniform contrast enhancement.

Similarly, Case 14 had a history of ataxia, aggressive behaviour, periodic spinal pain and lethargy and was referred for further investigations to the Neurology Department. At clinical examination, the dog had obtunded mentation, absent unilateral left menace response, pain on palpation of the temporal and cervical muscles and reduced paw replacement and hopping on the left thoracic and pelvic limbs consistent with chronic, progressive, painful right‐sided forebrain disease. MRI investigations revealed a large right intraventricular cerebral mass lesion and secondary syringomyelia as well as bilateral marked enlargement and increased T2W signal of the zygomatic salivary glands that prolapse rostrally and have a marked contrast enhancement.

Case 16 was referred to the Internal medicine department following a 24‐hour history of reverse sneezing/choking episodes. The dog underwent a CT scan, which revealed tonsillitis, left rhinitis and nasopharyngitis as the cause of the reverse sneezing. Additionally, the CT scan also revealed slight left unilateral exophthalmos, bilateral zygomatic gland enlargement with hypoattenuating contrast‐enhancing regions and increased contrast enhancement of the zygomatic glands. Furthermore, there was also generalized mild cranial/cervical lymphadenopathy.

### Haematological and biochemical findings

Haematology, venous blood gases and/or serum biochemistry were performed in 18/20 cases. Biochemical and haematological abnormalities were present in most cases tested (12/18), while electrolyte changes were present in 5/18 cases (Table 2). Neutrophilia (9/18) and leukocytosis (7/18) were the most common haematological abnormalities (Table 2). More common biochemical changes included increased alkaline phosphatase (4/18), alanine aminotransferase (3/18) and aspartate (3/18) and both hypoproteinaemia and hypoalbuminaemia (3/18) (Table 2).

**Table 2:** Haematological and biochemical changes reported in 18/20 dogs diagnosed with bilateral zygomatic sialadenitis

Haematological changes (number of affected dogs/total number of dogs tested)	Biochemical changes (number of affected dogs/total number of dogs tested)	
Neutrophilia (9/18)	Increased alkaline phosphatase (4/18)	Hyperphosphatemia (2/18)
Leukocytosis (7/18)	Increased alanine aminotransferase (3/18)	Increased creatine kinase (2/18)
Lymphopaenia (2/18)	Increased aspartate (3/18)	Increased urea (2/18)
Lymphocytosis (2/18)	Hypoproteinaemia and hypoalbuminaemia (3/18)	Parathyroid hormone (1/18)
Eosinopaenia (2/18)	Increased creatinine (2/18)	Increased amylase and lipase (1/18)
Monocytosis (1/18)	Hyperkalaemia (2/18)	
Monocytopaenia (1/18)	Hyperchloraemia (2/18)	

### Diagnostic imaging findings

Head MRI was performed in 12 cases (12/20 dogs, 24 glands) and CT in eight cases (8/20 dogs, 16 glands Table 3). The zygomatic glands parenchyma in the pre‐contrast study was heterogeneous in most cases (30/40). In the heterogeneous cases, fluid‐filled areas within the parenchyma were occasionally observed (5/20). In both MRI and CT studies (Table 3), contrast enhancement was mostly heterogeneous (30/40 glands) and fewer cases were homogeneous (10/40 glands).

**Table 3:** The computed tomography (CT) and magnetic resonance imaging (MRI) characteristics of the zygomatic glands in the 20 dogs diagnosed with bilateral zygomatic sialadenitis

Case	Shape		Type of imaging	MRI signal intensity				Homogeneity of the gland parenchyma		Homogeneity of contrast uptake		Symmetrical enlargement of the glands yes/no	Cellulitis yes/no	Myositis yes/no
	Left gland	Right gland		Left gland		Right gland		Left gland	Right gland	Left gland	Right gland			
				T2W	T1W	T2W	T1W							
1	Ovoid	Ovoid	MRI	Hyper	Hyper	Hyper	Hyper	Heterog	Heterog	Heterog	Heterog	Yes	Yes	Yes
2	Ovoid	Pyramidal	MRI	Hyper	Iso	Hyper	Hyper	Heterog	Homog	Ring‐like	Homog	No	Yes	Yes
3	Ovoid	Ovoid	MRI	Hyper	Hyper	Hyper	Hyper	Heterog	Heterog	Heterog	Homog	Yes	Yes	Yes
4	Ovoid	Ovoid	MRI	Hyper	Hyper	Hyper	Hyper	Heterog	Heterog	Ring‐like	Heterog	Yes	Yes	Yes
5	Pyramidal	Pyramidal	MRI	Hyper	Hyper	Hyper	Hyper	Homog	Homog	Homog	Homog	Yes	No	No
6	Pyramidal	Pyramidal	MRI	Hyper	Hyper	Hyper	Hyper	Heterog	Heterog	Heterog	Heterog	Yes	No	No
7	Pyramidal	Pyramidal	MRI	Hyper	Hyper	Hyper	Hyper	Homog	Homog	Heterog	Heterog	Yes	Yes	Yes
8	Tear‐drop	Pyramidal	MRI	Hyper	Hyper	Hyper	Hyper	Homog	Heterog	Heterog	Heterog	Yes	Yes	Yes
9	Ovoid	Ovoid	MRI	Hyper	Hyper	Hyper	Hyper	Heterog	Heterog	Heterog	Heterog	No	Yes	Yes
10	Ovoid	Ovoid	MRI	Hyper	Iso	Hyper	Hyper	Heterog	Heterog	Heterog	Heterog	No	Yes	Yes
11	Pyramidal	Pyramidal	CT	–	–	–	–	Heterog	Heterog	Heterog	Heterog	Yes	Yes	No
12	Pyramidal	Ovoid	CT	–	–	–	–	Heterog	Heterog	Heterog	Heterog	No	Yes	No
13	Pyramidal	Pyramidal	CT	–	–	–	–	Heterog	Heterog	Heterog	Heterog	Yes	Yes	No
14	Pyramidal	Pyramidal	MRI	Hyper	Hyper	Hyper	Hyper	Heterog	Heterog	Heterog	Heterog	No	Yes	Yes
15	Ovoid	Pyramidal	CT	–	–	–	–	Heterog	Heterog	Heterog	Homog	No	Yes	No
16	Pyramidal	Pyramidal	CT	–	–	–	–	Heterog	Heterog	Heterog	Heterog	No	Yes	No
17	Ovoid	Ovoid	CT	–	–	–	–	Homog	Homog	Heterog	Heterog	Yes	Yes	No
18	Pyramidal	Ovoid	CT	–	–	–	–	Heterog	Heterog	Heterog	Heterog	No	Yes	Yes
19	Ovoid	Pyramidal	CT	–	–	–	–	Heterog	Heterog	Heterog	Heterog	No	Yes	Yes
20	Ovoid	Ovoid	MRI	Hyper	Hyper	Hyper	Hyper	Homog	Homog	Heterog	Heterog	Yes	No	No

Heterog Heterogeneous, Homog Homogenous, Hyper Hyperintense, Iso Isointense

**FIG 6:**
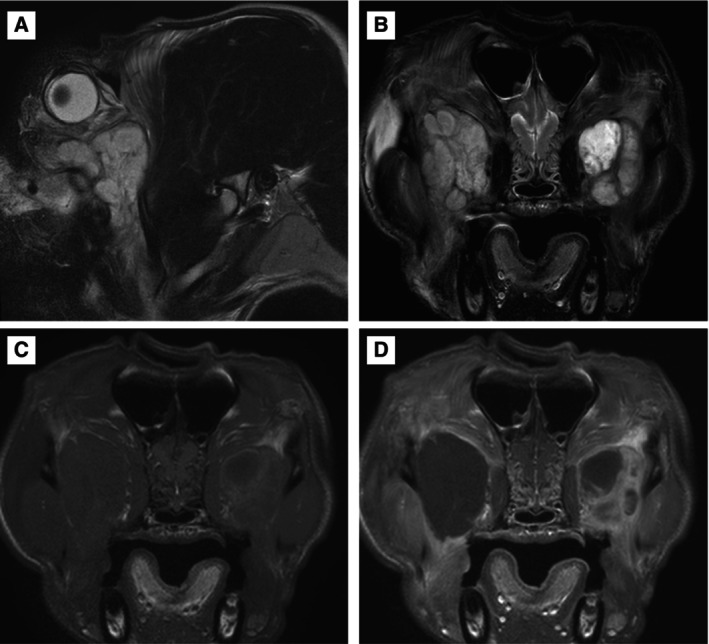
MRI studies obtained at the level of the zygomatic glands in a 7‐year‐1‐month‐old male entire Staffordshire Bull terrier with bilateral exophthalmos, pain on palpation of the head and on opening of the mouth (Case 10). (A) right oblique T2W, (B) transverse T2W, (C) transverse T1W and (D) T1W post‐contrast images. Note the marked enlargement of the right zygomatic salivary gland (A–D) with marked hyperintense signal on T2W images (A–C) when compared to the normal masticatory muscles. Within the medial half of the right gland, there is a lobulated area that appears hypointense on T1W (D) with thin contrast rim enhancement. The left zygomatic gland appears enlarged with lobulated appearance and moderate hyperintense signal on T2W (B–D). On T1W, the left gland appears isointense to the masticatory muscles with a thin contrast‐enhancing rim (D). The masticatory muscles show varying degrees of T2W hyperintensity suggesting cellulitis on both sides. Subcutaneous tissue swelling is also noted on the right side of the head.

**FIG 7:**
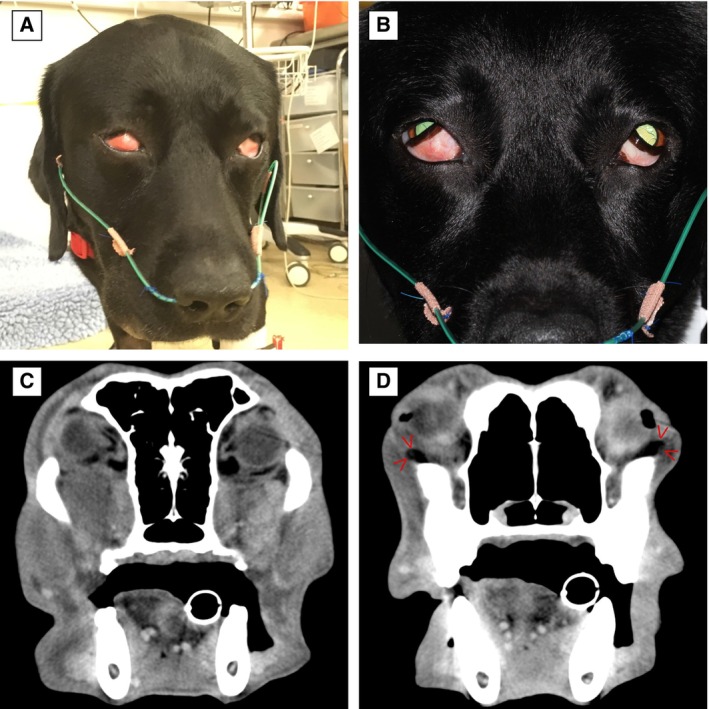
Six‐year‐4‐month‐old male neutered Labrador retriever (Case 12) at presentation (A) with sudden onset marked bilateral third eyelid protrusion, conjunctival hyperaemia, soft tissue swelling ventral to the lower eyelids and exophthalmos. (B) Third eyelid protrusion and conjunctival hyperaemia at 24‐hours of treatment. (C, D) Post‐contrast soft tissue window computed tomography images showing enlarged zygomatic glands and a mass effect owing to the anterior prolapse of the orbital fat (D, red arrowheads).

The affected glands (Fig 1) were classed as ovoid (20/40) or pyramidal (20/40) with a clearly defined contour (40/40) and appeared predominantly hyperintense on both T1 and T2‐weighted images (22/24 glands). Two left zygomatic glands (Cases 2 and 10, Fig 6) were isointense on T1‐weighted images when compared to the masticatory muscles. Symmetrical enlargement of the glands was observed in 10/20 cases. Surrounding cellulitis and myositis (of the temporal and masseter muscles) were found in 17/20 and 11/20 cases (Figs 1, 2 and 6) respectively. Retropharyngeal and/or mandibular lymph nodes appeared reactive in 9/20 cases. Retrobulbar fat displaced rostrally under the lower eyelids (Figs 4 and 7) was reported in 3/20 cases (Cases 12 to 14).

### Pathological findings

Cytological analysis was performed in 11/20 cases (Table 4). Samples were retrieved either by ultrasound‐guided fine needle aspiration (6/11) or via oral drainage (5/11) through the pterygopalatine fossa behind the caudal molar (Fig 5). Cytology results (Table 4) were non‐diagnostic in 2/11 cases and in the rest were consistent with degenerate neutrophils (9/11) with necrosis (2/11), mononuclear cells (1/11), intracellular cocci (3/11) and rod‐shaped bacteria (1/11).

**Table 4:** Results of the cytology and culture and sensitivity, type of medical treatment, length of treatment medication that was prescribed for when available and time until resolution of the clinical signs in 20 dogs diagnosed with bilateral zygomatic sialadenitis

Case	Cytology results	Culture and sensitivity performed yes/no	Anti‐microbial treatment, length of treatment medication was prescribed for	Anti‐inflammatory treatment, length of treatment medication was prescribed for	Others, length of treatment medication was prescribed for	Time to improvement/follow‐up	Euthanasia
1	Necrotizing sialadenitis	No	Amoxicillin clavulanate, 2 weeks	Non‐steroidal, 2 weeks	Omeprazole, 2 weeks	24 hours improved 11 days follow‐up resolved	–
2	–	Yes, no growth	Amoxicillin clavulanate, 4 weeks Metronidazole, 4 weeks	Non‐steroidal, 4 weeks	Fucithalmic^®^	24 hours improved 13 days follow‐up resolved	–
3	–	No	Amoxicillin clavulanate, 2 weeks	Prednisolone, 10 days	Benazepril, 1 month Amlodipine	24 hours improved 11 days follow‐up resolved	Yes
4	Neutrophilic suppurative inflammation, numerous cocci and chronic haemorrhage	No	Amoxicillin clavulanate, 2 weeks Metronidazole, 1 week	Non‐steroidal, 2 weeks	Omeprazole, 2 weeks Maropitant	48 hours improved 11 days follow‐up resolved 2 years follow‐up no recurrence	–
5	–	No	Amoxicillin clavulanate Metronidazole	–	Methadone Maropitant Allopurinol Benazepril	Eating well at 3 days No follow‐up	–
6	–	No	Amoxicillin clavulanate, 1 week	Non‐steroidal discontinued after 4 days due to melena	Metoclopramide Sucralfate Omeprazole	Eating well at 8 days 16 days follow‐up resolved	–
7	–	–	Clindamycin, 1 week	Non‐steroidal, 1 week	–	12 hours improved, no follow‐up	–
8	Non‐diagnostic	Yes, no growth	Amoxicillin clavulanate Metronidazole	–	Buprenorphine Chlorpheniramine Pantoprazole Methadone Paracetamol Fusidic acid Chloramphenicol	–	Yes
9	Blood contamination	Yes, no growth	Amoxicillin clavulanate, 2 weeks	Prednisolone, 1 month	Omeprazole, 1 week Methadone Chloramphenicol Serum Celluvisc^®^	24 hours improved 22 days follow‐up OS third eyelid gland prolapse	–
10	Neutrophilic inflammation, degenerated, bacteria, mononuclear cells, fibroplasia	Yes, *Escherichia coli* profuse growth	Amoxicillin clavulanate, 3 weeks Metronidazole, 3 weeks	Non‐steroidal	Omeprazole Isathal^®^	Enucleation OD 12 days follow‐up OS resolved 61 days follow‐up no recurrence	–
11	Neutrophilic inflammation with cocci	No	Amoxicillin clavulanate, 1 week	Non‐steroidal, 1 week	Omeprazole Methadone Fucithalmic^®^	24 hours improved, no follow‐up	–
12	Neutrophilic inflammation	Yes, *Acinetobacter*, *Pasteurella*, *Staphylococcus* spp.	Clindamycin, 2 weeks	Prednisolone, 6 weeks	Omeprazole, 1 week Chlorpheniramine, ongoing Salbutamol, ongoing Ranitidine, ongoing Tramadol Gabapentin Pentoxifylline, ongoing Amlodipine, ongoing	24 hours improved 35 days follow‐up no recurrence, 9 months follow‐up haemorrhagic gastroenteritis, no recurrence	–
13	Degenerate neutrophilic inflammation	Yes, no growth	Amoxicillin clavulanate, 4 weeks Clindamycin, 4 weeks	–	Tramadol	24 hours improved, 48 hours resolved 2 years follow‐up no recurrence	–
14	–	No	–	–	Methadone	–	Yes
15	Degenerate neutrophils, rare lymphocytes and macrophages, several rods bacteria	Yes, no growth	Amoxicillin clavulanate, 2 weeks	–	Buprenorphine Methadone Celluvisc^®^	24 hours improved, no follow‐up	–
16	–	No	Amoxicillin clavulanate, 2 weeks	Prednisolone, 1 week	Buprenorphine Maropitant	4 days improved 1 week recheck resolved	–
17	–	No	Clindamycin	Prednisolone	Amlodipine Omeprazole Tramadol Chloramphenicol Lubrithal^®^	23 days follow‐up resolved Still blind	–
18	Neutrophilic inflammation	Yes, no growth	–	Prednisolone	Phenobarbital Chloramphenicol	Lost to follow‐up	–
19	Neutrophilic inflammation with small amount of necrosis	Yes, no growth	Amoxicillin clavulanate	–	Phenobarbital Assisted feeding	Lost to follow‐up	
20	–	No	Amoxicillin clavulanate, 1 week	Non‐steroidal, 1 week	Omeprazole Viscotears^®^ Paracetamol	Initial improvement, 10 days follow‐up developed systemic signs	Yes

Culture results of the samples collected following aspiration or drainage were available in 9/20 cases (Table 4). Seven of 9 cases yielded no bacterial growth, 1/9 case revealed growth of *Escherichia coli* only and the other a mixed growth of *Acinetobacter*, *Pasteurella* and *Staphylococcus* spp.

**FIG 8:**
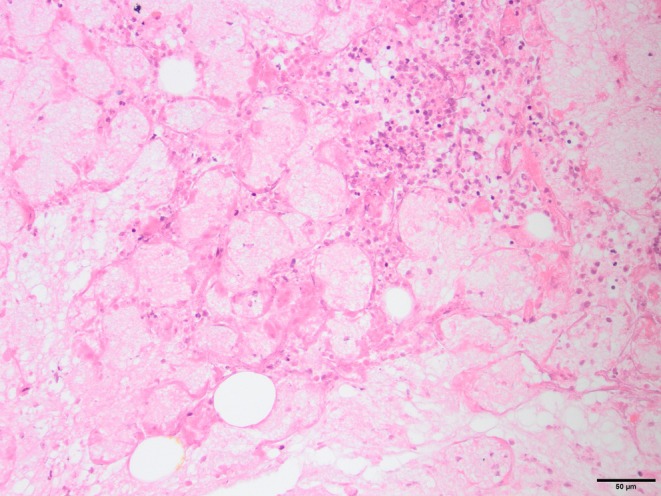
Histological image of the inflamed zygomatic gland biopsy retrieved during enucleation of Case 10. Note the presence of necrotic zygomatic gland acini undergoing coagulative necrosis, there is also infiltration of the tissue by a low number of neutrophils and macrophages (200×). Courtesy of Dr Simon Priestnall, Royal Veterinary College.

Histological analysis of the biopsied zygomatic gland was available for Case 10 and was consistent with salivary gland infarction and suppurative sialadenitis (Fig 8).

### Treatment and outcome

The treatment and outcome when available are detailed in Table 4. Antimicrobial treatment was administered in all but two cases and consisted of amoxicillin/clavulanate only (9/18), amoxicillin/clavulanate and metronidazole (5/18), clindamycin only (3/18) and amoxicillin/clavulanate and clindamycin (1/18). Where this information was recorded (in 14/18 dogs treated with antimicrobials, Table 4), antimicrobial medication was commonly prescribed for 2 weeks in 7/14 dogs, followed by 1 week course of medication in 4/14 dogs and 4‐week course treatment in 3/14 dogs (Table 4). Of the two cases that received no antimicrobial treatment, one dog was euthanised and the other had no response to treatment recorded. Eight of 20 dogs received systemic non‐steroidal anti‐inflammatory medication, 6/20 dogs were prescribed corticosteroids, and the rest 6/20 dogs received no anti‐inflammatory medication. Where it was recorded (11/14 dogs treated with anti‐inflammatory medication, Table 4), 6/14 dogs were prescribed a 1 week course of treatment, 3/14 and 2/14 dogs were treated for four to 6 and 2 weeks, respectively. Of the cases that received no anti‐inflammatory medication (6/20), 2/6 dogs were euthanised due to a diagnosis of sepsis and neoplasia, respectively; 3/6 were reported to have improved clinical signs and 1/6 had no response to treatment recorded.

Other medication (Table 4) administered as supportive treatment in 16/20 dogs more commonly included opioids (10/16), omeprazole (9/16), anti‐hypertensive medication (5/16), anti‐sickness medication (4/16), paracetamol/gabapentin (3/16) and chlorpheniramine (2/16). Phenobarbital was used in two cases with confirmed sialadenitis on cytology (Cases 18 and 19).

Of the three cases (Cases 2, 9 and 14) that were unilaterally blind at presentation, two (Cases 2 and 9) regained vision following treatment, whereas Case 14 was subsequently euthanised due to neoplasia (right‐sided intraventricular mass). One dog (Case 10) bilaterally blind on presentation regained vision after the orbital swelling responded to treatment; however, the dog subsequently developed right‐sided facial paresis during the hospitalisation and the right cornea became desiccated, requiring enucleation. His contralateral eye was diagnosed with vitreal haemorrhages that were reported to have resolved at the 6‐month follow‐up. One case (Case 17) that was visual at presentation lost vision during the hospitalisation due to suspected orbital compartment syndrome, which did not resolve following treatment. In total, 4/20 dogs (Cases 2, 9, 10 and 17) were reported to have developed suspected orbital compartment syndrome due to BZS. Four dogs (six eyes, Cases 8, 9, 10 and 17) were diagnosed with ulcerative keratitis likely secondary to corneal exposure (exposure keratitis) caused by the exophthalmos, and all but one eye (Case 10) resolved with medical treatment. Seven of 20 dogs received topical ocular antibiotic treatment, either chloramphenicol or fusidic acid (Table 4).

Clinical signs were reported to have improved in 16/20 dogs before they were discharged at home, with 10/16 cases improving within 48 hours and 2/16 dogs (Cases 5 and 6) reported to be eating well at Days 3 and 8 of treatment, respectively (Table 3). Eight of 20 cases had no follow‐up after their diagnosis and discharge from the hospital and, in those cases where it was recorded (12/20 dogs), the median follow‐up was 19 days (minimum of 10 days to maximum 2 years) with improvement in all but one case (enucleation of the left eye in Case 10) and no disease recurrence at their last follow‐up. Two cases (Cases 18 and 19) had no response to treatment recorded and no follow‐up after the dogs were discharged at home. Cases 8, 14 and 20 (Table 4) were euthanised due to grave systemic disease (duodenal foreign body with sepsis, intraventricular neoplasia and lymphoma stage V, respectively) soon after diagnosis, whereas Case 3 was euthanised 7 months later due to acute renal injury and hyperparathyroidism. Although later euthanised, the ocular signs of Case 3 improved after 24 hours of medical treatment with amoxicillin‐clavulanate, prednisolone, benazepril and amlodipine and had no recurrence of BZS at 1 month follow‐up. Before they were euthanised, Cases 8 and 20 also showed an initial improvement of the ocular signs at the 24‐hour and their 10‐day follow‐up examination, respectively.

## DISCUSSION

This is the first retrospective study investigating BZS in dogs. In the current study, the presumptive diagnosis of BZS was based on the clinical signs at presentation, imaging features, gland cytology results when available and/or response to treatment. Previously, only nine cases of BZS were reported in the veterinary literature (Bailey et al., 2021; Cannon et al., 2011; Fischer et al., 2019; Martinez et al., 2018; McGill et al., 2009; Winer et al., 2018). Based on the results of the present study, middle‐aged dogs appear to be more commonly affected by the disease, which is in line with the previous reports in unilaterally affected dogs (Cannon et al., 2011; Martinez et al., 2018). Labrador Retrievers represented half of the study population in the current study. No breed predisposition has been determined for unilateral zygomatic sialadenitis so far.

Corneal ulceration and blindness are reported complications of orbital disease resulting in exophthalmos. In the present study, four dogs were reported to be blind at presentation (three unilaterally blind and one bilaterally blind) likely owing to orbital compartment syndrome in three dogs and intraventricular neoplasia in one dog. Orbital compartment syndrome has been previously described in two dogs (Sauvage et al., 2018) but likely occurs with severe orbital and periorbital swelling whereby the blindness either results from a direct compressive effect on the optic nerve axons, reduction in its blood supply, or from a combination of the above. Of the three dogs that presented with unilateral blindness, two regained vision following treatment, and the other was euthanised. There are very few publications reporting vision loss after acute orbital disease, and none report recovery of vision following treatment, and this could be attributed to the delay in referring the patient and prompt diagnosis and treatment. Interestingly, Case 17, which was visual on initial presentation, was reported to have lost vision in both eyes despite the medical treatment.

Corneal exposure is a reported complication of enlarged globes (buphthalmos), proptosis, exophthalmos, severe orbital/periorbital or eyelid swelling and facial nerve paralysis/paresis with impaired blink. Corneal exposure may be aggravated by restricted eyelid movement, reduced corneal sensitivity secondary to severe orbital swelling and systemic use of opioids for analgesia known to reduce tear production, all these factors predisposing to corneal ulceration. It is therefore important to provide topical lubrication in cases of exophthalmos or with severe periorbital swelling while pending their diagnosis and treatment. The cases diagnosed with corneal ulceration were all likely secondary to exposure keratitis associated with the exophthalmos and/or severe periorbital swelling as they were diagnosed on presentation. They were treated with topical antimicrobial and lubrication drops, and all but one resolved within the course of their hospitalisation stay. Only one case (Case 10) underwent enucleation due to no improvement in the clinical signs and severely desiccated cornea despite the medical treatment likely aggravated by the dog developing facial nerve paresis during hospitalisation.

There were also three cases with no reported ocular or periorbital signs prior to referral where BZS was incidentally diagnosed following advanced imaging. Two of these cases were referred to the Internal medicine department for acute vomiting and generalised ataxia and sneezing/choking episodes, respectively. The other case presented to the Neurology department with a history of periodic spinal pain and ataxia. Two cases underwent MRI due to abnormal neurological findings, and the third one underwent a CT scan to investigate the upper airways. Two of these cases were reported to be painful on palpation of the head. An explanation for the lack of BZS‐associated clinical signs recorded could be that the pain was attributed to other underlying diseases rather than BZS, incomplete clinical records, incomplete physical examinations, or lack of specialist ophthalmic examination. One of the dogs did have signs of exophthalmos on the cross‐sectional imaging studies but was not reported clinically. It is possible that there was mild clinical exophthalmos thought to be breed‐related as the dog was of a brachycephalic breed. The exophthalmos would likely cause more pain in dolichocephalic dogs with tight eyelids compared to brachycephalic dogs with a shallow orbit; therefore, they may not exhibit signs of pain early in the course of the disease, thus being missed by the owners or on initial examination.

In the present study, cytological and histopathological analysis of the glandular tissue was available for just over half of the cases, revealing predominantly neutrophilic inflammation. The cases where cytology or histopathology analysis was not performed were included in the study based on the clinical and imaging findings suggestive of sialadenitis, such as painful bilateral zygomatic gland enlargement and contrast enhancement with surrounding inflammation (of the temporal and masseter muscles), respectively, as well as the response to treatment. The cases that were treated with phenobarbital were included in the study as they had a cytological diagnosis consistent with sialadenitis.

A definitive diagnosis of this condition would ideally be based on cytological, histological, or culture and sensitivity results. However, due to the retrospective nature of this study, the response to antimicrobial and anti‐inflammatory treatment, the clinical findings consistent with painful disease, or evidence of adjacent inflammation (such as temporal and masseter muscle inflammation) on the MRI or CT images were utilised as means to include the cases in the present study population. This may be considered a limitation of this study, as a definitive diagnosis was never really reached for some of these cases. Furthermore, sampling orbital disease may be challenging, with cytological samples poorly exfoliative and non‐diagnostic. Moreover, in some cases, management of their concurrent or underlying systemic disease was prioritised over the diagnosis of BZS; hence, further sampling of the glands may not have been considered.

Treatment for the dogs in the present study mostly consisted of systemic antimicrobial, anti‐inflammatory medication and supportive treatment for their underlying systemic disease. This is in line with the literature published on unilateral zygomatic sialadenitis. Most dogs were treated with antimicrobial treatment for at least 2 weeks and anti‐inflammatory medication for at least 1 week, with more than half of the dogs (16/20 dogs) showing improvement in the clinical signs before they were discharged from the hospital or before they were euthanised. Of the 12/20 cases that had a follow‐up after their diagnosis, none had BZS recurrence at their last examination.

Phenobarbital has been reported as the sole treatment for sialadenitis in a dog with BZS non‐responsive to antimicrobials (Martinez et al., 2018) and as adjunctive treatment, alongside antimicrobials or anti‐inflammatory medication in dogs with salivary gland necrosis (Schroeder & Berry, 1998). In the present study, two dogs also received oral phenobarbital alongside antimicrobial or anti‐inflammatory treatment; however, with no documented response to treatment.

Normal zygomatic glands have been characterised on MRI and CT in several studies (Durand et al., 2016; Gil et al., 2018; Weidner et al., 2012). Signal intensity of the normal salivary glands appears to be isointense to hyperintense compared to muscular tissue on T1‐weighted images and hyperintense on T2‐weighted images, based on a study by Weidner et al. (2012). In a cadaveric study by Gil et al. (2018), the normal glands were hyperintense in comparison to the osseous and muscular tissues, heterogeneous and the most consistent anatomical landmark was the pterygopalatine fossa.

MRI has been advocated as the gold standard imaging modality for the evaluation of salivary gland diseases (Durand et al., 2016; Gil et al., 2018). T2‐weighted images appear to give a better definition of the glands and surrounding structures relative to T1‐weighted images, according to a study evaluating normal salivary glands in canine cadavers (Gil et al., 2018). Cannon et al. (2011) have shown that the MRI findings were characteristic of sialadenitis as the affected glands appeared enlarged, hypointense on T1‐weighted, hyperintense on T2‐weighted and presented increased contrast enhancement compared to masticatory muscles signal (Cannon et al., 2011). In clinical settings, there are practical limitations of MRI, such as cost, availability and longer scan times compared to CT. Despite the superior soft tissue resolution and ability to differentiate between normal and pathological tissue seen with MRI, other imaging techniques such as CT are appropriate and more often used for the diagnosis of orbital and salivary gland disease with a sensitivity of 57% and specificity of 90% for CT diagnosis of inflammatory zygomatic salivary gland enlargement (Winer et al., 2018).

In the present study, the glands were enlarged and mostly hyperintense on both T1 and T2‐weighted MRI studies, in accordance with the literature. Furthermore, they presented heterogeneous contrast enhancement throughout the parenchyma. On pre‐contrast CT images, the glands appeared hypoattenuating and had heterogeneous contrast enhancement with occasional hypodense areas within the parenchyma, presumed necrotic hypovascularized areas or mucoceles; regardless, these areas should be avoided for sample collection. Perilesional myositis, cellulitis, regional lymphadenomegaly and mass effect with retrobulbar fat displaced rostrally above the ventral orbital rim were also common findings in this study.

Concurrent systemic disease was present in most dogs in this study, with allergic skin and gastrointestinal disease being most prevalent. Affected dogs also commonly presented with gastrointestinal signs of either vomiting, diarrhoea, or inappetence before the ocular signs developed. Kidney disease, hypertension and pancreatitis were also reported in a few cases. Previously reported systemic signs or diseases in dogs with zygomatic sialadenitis (unilateral or bilateral) included granulomatous dermatitis, lethargy, anorexia, fever and diabetes ketoacidosis (Bailey et al., 2021; Cannon et al., 2011). One case report described BZS developing in the postoperative period after exploratory laparotomy and gastrointestinal foreign body removal (Martinez et al., 2018). In the present study, two dogs had duodenal foreign bodies diagnosed, one of no clinical significance and the other being subsequently euthanised due to secondary sepsis.

Interestingly, in people, acute sialadenitis has been described to typically affect one major salivary gland (typically the parotid gland) and it is more commonly seen in medically debilitated, hospitalised or postoperative patients (Koizumi et al., 2015; Uchino et al., 2016; Watanabe et al., 2018; Yim et al., 2017). Retrograde bacterial contamination from the oral cavity has been suggested as the inciting aetiology due to stasis of the salivary flow secondary to dehydration or decreased oral intake (Abdel Razek & Mukherji, 2017; Thomas et al., 2010). Gastrointestinal signs and anorexia/hyporexia were commonly reported in this study; this may explain the development of BZS in dogs with systemic diseases, dehydrated, or anorexic dogs in this study. Diabetes mellitus, hypothyroidism, renal failure and Sjögren's syndrome are among the predisposing factors for acute sialadenitis in people (Mandel, 2014; Thomas et al., 2010). Similarly, in the present study, three dogs had kidney disease, one dog had diabetes and another one had hypothyroidism. Inflammation of the salivary glands has also been suggested as a possible consequence of chronic nausea, regurgitation, or vomiting associated with primary gastrointestinal disease in people (Sato & Fukudo, 2015).

In addition, several systemic conditions have been associated with salivary gland disease in people, such as immunoglobulin‐G4‐related sialadenitis (Abdel Razek & Mukherji, 2017; Vasaitis, 2016), human immunodeficiency virus (HIV)‐sialopathy, Influenza A‐associated sialadenitis (Mandel, 2014; Stafford et al., 2018), Sjögren's sialadenitis (Abdel Razek & Mukherji, 2017) and bilateral parotid sialadenosis in alcoholics, diabetics and malnourished patients (Garcia Garcia et al., 2018; Mandel & Hamele‐Bena, 1997). Further research, such as histopathology and immunohistochemistry, would be required to characterise the pathogenesis of BZS in dogs.

There are several limitations that need to be considered when interpreting the results of the present study, including the retrospective nature of the research that limits the consistency and standardisation of medical records search and data collection (the lack of ophthalmic examination and incomplete follow‐up in some cases) across the three referral centres. The retrospective search was performed at different time points: December 2018 at the AHT before its closure and December 2019 at the RVC and LVH. The author first conducted the study at the AHT, then relocated to the RVC, where the study was re‐started and LVH were invited to collaborate in order to increase the sample size. This explains the difference in the time points for the medical records search. The selection of CT *versus* MRI scan was determined by the owner's finances or the clinician's preference due to a shorter time for image acquisition for CT *versus* MRI. The small number of cases included is another factor limiting the statistical power to generalise findings; it does, however, reflect the infrequency of the condition. As the total length of treatment was not recorded for all the cases, it makes it challenging to evaluate the consistency or adequacy of therapeutic approaches across the study population. The incomplete/absent follow‐up also limits the ability to assess long‐term outcomes, recurrence rates and the full effectiveness of the treatments used.

This retrospective observational study provides valuable insights into the clinical presentation, imaging features and outcomes of dogs with bilateral zygomatic sialadenitis. Key findings include the frequent association with systemic diseases, which may play a role in the pathogenesis of the condition. Additionally, the study highlights the importance of associating the imaging, cytological and histopathological results with the clinical examination findings. There is a generally favourable response to systemic antibacterial and anti‐inflammatory treatments as well as supportive treatment for the concurrent disease when present. MRI features characteristic of BZS are bilaterally enlarged glands, hyperintense on T1 and T2‐weighted images compared to masticatory muscles and hyperattenuating with contrast enhancement on CT images. The aetiopathogenesis of BZS remains unknown and further research is required.

### Author contributions

**A. E. Enache:** Conceptualization (equal); data curation (lead); formal analysis (equal); investigation (lead); methodology (supporting); writing – original draft (lead). **S. Maini:** Data curation (equal); formal analysis (equal); supervision (equal); writing – review and editing (equal). **M. Pivetta:** Conceptualization (equal); data curation (equal); formal analysis (lead); writing – review and editing (equal). **E. Jeanes:** Data curation (equal); writing – review and editing (equal). **L. Fleming:** Data curation (supporting); writing – review and editing (equal). **C. Hartley:** Formal analysis (equal); supervision (equal); writing – review and editing (equal). **R. Tetas Pont:** Conceptualization (lead); formal analysis (equal); methodology (lead); supervision (lead); writing – review and editing (equal).

### Conflict of interest

None of the authors of this article has a financial or personal relationship with other people or organisations that could inappropriately influence or bias the content of the paper.

## Data Availability

The data that support the findings of this study are available on request from the corresponding author. The data are not publicly available due to privacy or ethical restrictions.
